# Microfluidic isolation and release of live disseminated breast tumor cells in bone marrow

**DOI:** 10.1371/journal.pone.0319392

**Published:** 2025-03-12

**Authors:** Minh-Chau N. Le, Dongjiang Chen, Kierstin A. Smith, David D. Tran, Z. Hugh Fan

**Affiliations:** 1 Department of Mechanical and Aerospace Engineering, Interdisciplinary Microsystems Group, Gainesville, Florida, United States of America; 2 Division of Neuro-Oncology, USC Keck Brain Tumor Center, University of Southern California Keck School of Medicine, Los Angeles, California, United States of America; 3 J. Crayton Pruitt Family Department of Biomedical Engineering, GainesvilleFlorida, United States of America; The Ohio State University, UNITED STATES OF AMERICA

## Abstract

Breast cancer represents a significant therapeutic challenge due to its aggressive nature and resistance to treatment. A major cause of treatment failure in breast cancer is the presence of rare, low-proliferative disseminated tumor cells (DTCs) in distant organs including the bone marrow. This study introduced a microfluidic-based approach to improve the immunodetection and isolation of these rare DTCs for downstream analysis, with an emphasis on optimizing immunocapture, release, and enrichment methods of live DTCs as compared to the standard approach for blood-borne circulating tumor cells (CTCs). EGFR (epidermal growth factor receptor) and EpCAM (epithelial cell adhesion molecule), two key cell surface markers in breast cancer, were validated as efficient cell capture targets for DTCs within microfluidic chambers. Furthermore, we demonstrated that a combination of 0.25% trypsin and impulse was the most effective for releasing captured cells, maintaining high viability, and preserving essential cellular characteristics. Using a metastatic mouse breast cancer model, we achieved a 47.9-fold enrichment of live DTCs. Analysis of blood and bone marrow samples obtained from a breast cancer patient with minimal residual disease at two timepoints revealed a reduction in CTCs and an increase in DTCs following adjuvant chemotherapy. This observation suggested a potential dynamic interplay between CTCs and DTCs in response to therapy. Our results underscore the potential of the microfluidic approach in enhancing DTC detection and shed light on the importance of monitoring both CTCs and DTCs in breast cancer prognosis and treatment response assessment.

## Introduction

Breast cancer, the second-leading cause of cancer-related deaths for women in the U.S. [1], presents a significant challenge in managing minimal residual disease, particularly due to the persistence of tumor cells undetectable by standard diagnostics after therapy [2,3]. The presence of those elusive tumor cells, called disseminated tumor cells (DTCs), in the bone marrow (DTC-BMs) has been linked to increased relapse risk and poor prognosis [4,5]. Thus, there are urgent needs to understand and develop novel therapeutics targeting DTC-BMs [6,7]. Recent work has identified a set of genes (EpCAM, GLI3 etc.) based on DTC-BM utilizing high-throughput, quantitative reverse transcription polymerase chain reaction (qRT-PCR) for predicting metastatic relapse [8]. However, this is still not sufficient for studying the features of DTC-BM comprehensively.

The isolation and study of DTCs pose significant challenges due to their rarity and the invasive procedures required to obtain bone marrow (BM) samples. These difficulties are compounded by the need for methods that can accurately identify and isolate DTCs from a dense cellular environment [9]. Traditional techniques for enriching and capturing these cells are often labor-intensive [9], with low sensitivity [2] and purity [10], underscoring the need for innovative approaches.

Microfluidic technology, known for its precision in manipulating fluids at a microscopic scale, offers promising solutions for the detection and isolation of rare cells like circulating tumor cells (CTCs) [11,12]. This technology integrates various functionalities into a single platform, allowing for enhanced control and efficiency. However, the application of microfluidics in isolating DTCs-BM has been relatively unexplored compared to its use with CTCs. Recently, several groups have adopted the filtration-based Parsortix® microfluidic system for the enrichment of DTCs in the BM of breast cancer patients [13,14].

Herein, we report advances in utilizing an immunoaffinity-based microfluidic device [15] for DTC detection. Our study explores various antibodies for high-efficiency capture of DTCs, ensuring the preservation of cell viability, morphology, and surface expression integrity essential for downstream analyses. Employing a mouse DTC model, we demonstrate the device’s efficacy in enriching DTCs for single-cell assays and present a novel comparison of DTC-BM detection to CTC enumeration in a breast cancer patient. Note that there are a large body of literature on using microfluidics for CTC isolation, but there is little work of using microfluidics on DTC analysis. The most of DTC research were performed using CellSearch or standard biological assays [4,5].

## Materials and methods

### Reagents and buffers

Biotinylated antibody against epithelial cell adhesion molecule (anti-EpCAM) (eBioscience, Carlsbad, CA, USA) and biotinylated antibody against epidermal growth factor receptor (anti-EGFR, Research Grade Cetuximab Biosimilar) (R&D Systems, Minneapolis, MN, USA) were used as the tumor cell capture agents immobilized on the surface of the microchannels. Human TruStain FcX Fc Receptor was purchased from BioLegend (San Diego, CA, USA). Cell dissociation reagent 0.25% trypsin-ethylenediaminetetraacetic acid (trypsin-EDTA, 1X, phenol red) was purchased from Caisson Labs (Smithfield, UT, USA). Cell dissociation reagent 2.5% trypsin (10X, no phenol red) was obtained from Gibco (Scotland, UK). A pan-reactive antibody against cytokeratin (panCK, AE1/AE3), conjugated with Alexa Fluor 488 (AF), was bought from eBiosciences (Carlsbad, CA, USA), while antibody against CD45, conjugated with phycoerythrin (PE) was from BD Biosciences (Franklin Lakes, NJ, USA). PanCK-AF and CD45-PE were used in immunocytochemistry staining to identify tumor cells and hematopoietic cells, respectively. An antibody against cytokeratin (CK, CAM 5.2), conjugated with fluorescein isothiocyanate (FITC) (BD Biosciences, Franklin Lakes, NJ, USA), was used as an alternative to panCK in the immunocytochemistry staining of some healthy BM samples. 4’,6-Diamidino-2-Phenylindole (DAPI, Dilactate) (Invitrogen, Waltham, USA) was used to stain the nucleated cells. Dulbecco’s phosphate buffered saline without calcium and magnesium (DPBS) (Cytiva, Marlborough, MA, USA) was used for washing during cell preparation, device functionalization, and sample processing. DPBS containing 2% bovine serum albumin (2% BSA-DPBS) (Fisher Scientific, Waltham, MA, USA) was used for cell dilution and blocking during device functionalization and immunocytochemistry staining. PE-labeled streptavidin (BioLegend, San Diego, CA, USA) and fixable viability dye eFluor 780 (eBioscience, Carlsbad, CA, USA) were used for flow cytometry. BD Pharmingen Purified Rat Anti-Mouse CD16/CD32 Fc Block (BD Biosciences, Franklin Lakes, NJ, USA) was used as the Fc blocking agent for mouse BM sample preparation. BD Pharm Lyse buffer (BD Biosciences, Franklin Lakes, NJ, USA) was used to deplete red blood cells in both human and mouse BM samples when necessary.

### Device fabrication and preparation

For this work, the previously reported microfluidic device based on geometrically enhanced mixing (GEM) (**S1****A Fig** in **Supplementary Information**) was used [15]. The device fabrication is briefly described here. First, from a 5” chrome photomask (Nanofilm, Westlake Village, CA, USA) imprinted with the device design, a silicon master containing two-layer GEM structures was produced via standard photolithography with SU-8 2035 (Kayaku Advanced Materials, Westborough, MA, USA) serving as the negative photoresist. Then, Dow Sylgard 184 polydimethylsiloxane (PDMS) (Ellsworth Adhesives, Germantown, WI, USA) was casted over the silicon master and cured at 60 °C for 3 hours to form a PDMS substrate embossed with the device features. Finally, the device features on the PDMS substrate were sealed with a 3-inch by 1-inch glass slide.

Prior to using the microfluidic device, antibodies were immobilized onto the microchannel surfaces using the following functionalization process. Ethanol (2 µL/s, 300 µL) was introduced into the microchannels of the PDMS device to decrease their surface tension, followed by a DPBS wash (2 µL/s, 900 µL). 100 µL of 2 mg/mL avidin (Invitrogen, Carlsbad, CA, USA) in DPBS was loaded into the device, incubated for 15 min at room temperature to allow for the physical adsorption of avidin onto the microchannel surfaces, followed by washing with DPBS (1 µL/s, 300 µL). Then, 100 µL of either 20 µg/mL biotinylated anti-EpCAM or 10 µg/mL biotinylated anti-EGFR in DPBS was introduced into the device, allowed to incubate for 15 min, followed by rinsing with 2% BSA-DPBS (1 µL/s, 300 µL). Finally, the device was incubated for 20 min with the 2% BSA-DPBS, which acted as a blocking buffer to reduce non-specific capturing of cells. Functionalized devices were stored in a foil-wrapped humidified petri dish at 4 °C until use.

### Culture of breast cancer cells

MDA-MB-231 (triple-negative, TN) and MCF-7 (estrogen-receptor-positive and progesterone-receptor-positive, ER^+^PR^+^) human breast cancer cells were purchased from ATCC (Manassas, VA, USA). Cells were cultured in T25 cell culture flasks in Dulbecco’s Modified Eagle’s Medium (DMEM) (Corning, Manassas, VA, USA) supplemented with 10% fetal bovine serum (FBS) (Corning, Manassas, VA, USA) and 1% penicillin-streptomycin (PS) (Sigma-Aldrich, St. Louis, MO, USA), and incubated at 37 °C with 5% CO_2_ in a humidified incubator. Cells were expanded to 80% confluency before being detached with 0.25% trypsin-EDTA for experiments or sub-culturing at a lower concentration. The media of cell cultures were changed every other day.

### Nested quantitative PCR

RNeasy Mini Kit (QIAGEN, Venlo, The Netherlands) was used to extract RNA from cells/tissues according to the manufacturer’s protocol. One µg total RNA was subjected to reverse transcription using iScript cDNA Synthesis Kit (BIO-RAD, Hercules, CA, USA). qPCR was performed using Luna Universal qPCR Master Mix (New England Biolabs, Ipswich, MA, USA) and on CFX96 Touch Real-Time PCR system from BIO-RAD. The optimized primary-amplification cycle number is 20 with an annealing temperature of 60°C, while second amplification is 40 cycles with 63°C as annealing temperature [16]. Primers used are as follow: KRT19 forward primer (fw): GCGAGCTAGAGGTGAAGATC, reverse primer (rev): ACTTGGTTCGGAAGTCATCTG; KRT19 (nested) fw: CCCGCGACTACAGCCACTAC, rev: AGACGGGCATTGTCGATCTG; EpCAM fw: GGACACTGAAATAACCTGCTC, rev: GGATCCAGTTGATAACGCG; EpCAM (nested) fw: CCTGCTCTGAGCGAGTGAGA, rev: TCTGAAGTGCAGTCCGCAAA; EGFR fw: CCAAGCCATATGACGGAATCC, rev: GGAACTTTGGGCGACTATCTG; EGFR (nested) fw: AAGGAGAACGCCTCCCTCAG, rev: TTGGGCGACTATCTGCGTCT; GLI3 fw: ACTCCTTGGTCACGATTCTC, rev: CGGAAGAGTAGGTGAAGCTC; GLI3 (nested) fw: TCCCGTAGCAGCTCTTCAGC, rev: AGCTCAAGGCAGGGCTGATT; MUC1 fw: TCCGAGAAGGTACCATCAAT, rev: AAAGGAAATGGCACATCACT; MUC1 (nested) fw: CGACGTGGAGACACAGTTCAA, rev: GGCACATCACTCACGCTGAC; IL6R fw: CAGTAGTGTCGGGAGCAAGT, rev: CTTGACCATCCATGTTGTGA; IL6R (nested) fw: TGTGGAATCTTGCAGCCTGA, rev: GTTCCAGGAGTGGGGGTCTT; GAPDH fw: CATCATCCCTGCCTCTACTG, rev: TTGGCAGGTTTTTCTAGACG; GAPDH (nested) fw: GGTCATCCCTGAGCTGAACG, rev: TCAGGTCCACCACTGACACG.

### Cell transfection

Green fluorescent protein (GFP) – transfected MDA-MB-231 (MDA-MB-231-GFP) cells were carried out in-house by stably infecting lentivirus into MDA-MB-231 cells. To produce lentivirus, we used polyethyleneimine (PEI, 1µg/µl, Sigma-Aldrich, St. Louis, MO, USA) at a 3:1 ratio of PEI (µg): total DNA in the pLL3.7 backbone (µg) to transfect HEK 293T cells. PSPAX2 and PMD2.G plasmids were used for viral packaging and enveloping, respectively in advanced DMEM media supplemented with 1.25% FBS, N-2-hydroxyethylpiperazine-N-2-ethane sulfonic acid (HEPES), pyruvate and sodium butyrate. After infection, GFP positive cells were then sorted by flow cytometry, with >90% positive rate in culture.

### Flow cytometry

Surface proteins on transfected and other cells were studied using flow cytometry. Single cell suspension was Fc-blocked before incubated with primary and fluorochrome-conjugated secondary antibodies for 20 min at 4^o^C in the dark. Biotinylated anti-EpCAM and anti-EGFR were used as primary antibodies, and streptavidin-PE was used as the detection moiety to label the biotinylated antibodies. A gate was set based on coordinated cells incubated with primary antibodies only. Flow cytometry was performed on a BD FACS Canto II and analyzed by FlowJo_V10 (BD). 488 nm blue laser and 585 nm emission filter/42 nm bandpass filter were used to detect PE signal. Live cells were separated from debris in an SSC-A (y) versus FSC-A (x) dot plot, doublets excluded with FSC-H (y) versus FSC-A (x)/ SSC-H (y) versus SSC-A (x) dot plots. Singlets were analyzed and gated as indicated. For mouse bone marrow samples enriched using GEM, depending on how many cells were released post microfluidic devices, at least 20,000 events were collected for analyzing. Three repeats were performed in each testing condition.

### Cell capture characterization

MDA-MB-231 cells were stained with either Hoechst 33342 fluorescence nucleic acid stain (ImmunoChemistry Technologies, Bloomington, MN, USA) or Vybrant^TM^ DiO cell-labeling solution (Invitrogen, Carlsbad, CA, USA) at 0.5% volume/volume. Then, stained cells were spiked into 2% BSA-DPBS at a concentration of 100-1,000 cells/mL. To ensure the accuracy of cell concentration, the final cell concentration was obtained via serial dilutions with dilution factors 1:10 or lower. The cell solution was introduced into the microfluidic devices (1 µL/s, 1 mL) previously functionalized with either anti-EpCAM or anti-EGFR antibodies. The general experimental setup for the introduction of samples (e.g., cell solution, clinical sample) and reagents into a microfluidic device was as described previously [17]. After cell introduction, the device was washed with DPBS (2 µL/s, 900 µL) to remove unbound cells. The capture efficiency was determined using the formula below.


$$Captureefficiency(\%)=\frac{Cells_{capturedindevice}}{Cells_{introducedintodevice}}\times 100$$

To study the effect of BM matrix, the cell solution of MDA-MB-231 cells stained with Hoechst or DiO at a concentration of 100-1,000 cells/mL was mixed with the healthy BM at a 1-to-1 ratio. The cell-BM mixture was introduced into a functionalized microfluidic device at 1 µL/s. After sample introduction, the device was washed with DPBS (2 µL/s, 900 µL) to remove unbound cells.

### Release of captured cells and their characterization

For characterizing the release of capture cells, GEM devices functionalized with 10 µg/mL anti-EGFR were used. The methods under investigation utilized chemical, mechanical, or a combination of both means to release cells. Trypsin was the chemical of interest. Impulses, created by periodically tapping on the PDMS substrate on top of the microchannels, were the mechanical forces (**S2 Fig**). The five cell release methods investigated were (1) 0.25% trypsin, (2) 2.5% trypsin, (3) impulse, (4) 0.25% trypsin with impulse, and (5) 2.5% trypsin with impulse. Trypsin was used at two concentrations: (1) 0.25%, a lower concentration widely used for releasing adherent cancer cells from culture surface for subculturing [18], and [2] 2.5%, a higher concentration used for the enzymatic digestion of both cells and tissues [19–21]. To release cells by chemical means, either 0.25% trypsin or 2.5% trypsin was added to the device via the inlet (1 µL/s, 150 µL). Then, the device was incubated at 37°C for 5 min to expedite cell detachment. Finally, fully supplemented cell culture media was added to the device via the outlet at a high flow rate (5 µL/s, 4 mL) to wash out the released cells. The flow was reversed for the wash step (i.e., flow going from the outlet to the inlet) due to the majority of the captured cells locating in the front half (i.e., near the inlet) of the device. To release cells via only mechanical forces, no trypsin was added to the device, and impulses were applied during the high flow rate wash step with cell culture media. Combinations of trypsin and impulse (i.e., 0.25% trypsin with impulse, and 2.5% trypsin with impulse) were also tried, which included the trypsin incubation step, the high-flow-rate media wash step, and the impulses. The cell release efficiency was calculated according to the formula below.


$$Releaseefficiency(\%)=\frac{Cellsindevice_{beforerelease}-Cellsindevice_{afterrelease}}{Cellsindevice_{beforerelease}}\times 100$$

After collecting released cells from devices by the three most efficient release methods (impulse, 0.25% trypsin with impulse, and 2.5% trypsin with impulse), viability and EGFR surface marker expression were assessed. The released cells were stained by biotinylated EGFR primary antibody followed by PE streptavidin and viability 780 dye (eBioscience, Carlsbad, CA, USA). Singlets were analyzed and gated as previously described [22]. Cells stained by viability 780 dye were excluded as dying cells, PE staining was utilized to indicate surface EGFR levels comparing under each condition.

### Culture and analysis of released cells

During the high-flow-rate media wash step of cell release, the product was collected from the inlet of the device into 15-mL centrifuge tubes. The collected cell solution was centrifuged at 200 × g for 5 min to collect the cell pellet. Then, cells were resuspended in fresh fully supplemented media and seeded in 96-well plates. For comparison, MDA-MB-231-GFP cells that were not subjected to microfluidic capture and release were also cultured as the controls. Cells were cultured for 5 days at 37 °C with 5% CO_2_ and the media was changed every two days.

At timepoints 1, 3, and 5 days, morphology and growth analyses were conducted on control and released cell cultures. To compare the morphology between the released cells and the control cells, cell cultures were imaged at 4X at various locations (n = 3) in each well. The Gen5 software was used to analyze the fluorescence images for circularity, a parameter representing the roundness of an object. Circularity was calculated based on the ratio between the area of a defined object in the image and the area of a circle with the same perimeter. A value of 1 denoted a perfect circle, while values less than and further away from 1 denoted non-circular shapes with decreasing roundness. For analysis of cell growth, cultures were imaged at 4X at the same location in each well at the three timepoints. The Gen5 software was used to determine the cell number from the images.

### Mouse DTC model

The relevant animal work protocol has been reviewed and approved by the University of Florida Institutional Animal Care and Use Committee (IACUC) under IACUC# 202209922. Mammary fat pad injection was performed to establish the tumor model. Mice were anesthetized with isoflurane, and a preemptive dose of meloxicam was given subcutaneously, followed by lidocaine. Ophthalmic ointment was applied to the eyes of the mice to prevent drying. One million MDA-MB-231-GFP cells were implanted into the mammary fat pad of immunocompromised NSG mice (Jackson Laboratory) and allowed to develop into primary and secondary metastatic tumors (S3B Fig). For control, DPBS was injected into the mammary fat pad of immunocompromised mice (S3A Fig).

After injection, regular chow was provided, and the mice were monitored daily. The designed endpoint was five to eight weeks post-injection for tissue collection. Mouse BM was collected by flushing out the content of the femurs. Tumor-bearing and control BM samples were prepared in 2% BSA-DPBS at a concentration of 10,000,000 cells/mL. This endpoint was set before any humane endpoints would be reached, which included ≥ 15% weight loss from baseline weight, a body condition score of 2 or less, inability to reach food or water, labored breathing, respiratory distress or cyanosis, and any tumor that became ulcerated or exceeded 1.5 cm in any dimension. A total of 15 mice were used, and all were euthanized immediately upon reaching the study endpoint by inhalation of carbon dioxide followed by cervical dislocation. No animals were lost before meeting the criteria for euthanasia.

### Enrichment of DTCs in mouse BM

For the enrichment of DTCs in BM, GEM devices functionalized with 10 µg/mL anti-EGFR were used. Three conditions with slightly different sample preparation and processing flow rates were tested: (1) normal flow rate, (2) Fc blocking, and (3) Fc blocking with adjusted flow rates (**S4****A Fig**). For normal flow rate, the BM sample was diluted 1:1 with 2% BSA-DPBS and introduced into the microfluidic device at 1 µL/s for cell capture. After cell introduction, like the steps described for cell capture experiments, the device was washed once with DPBS (2 µL/s, 900 µL). For Fc blocking, Fc blocking reagent was added to the BM sample to suppress the non-specific binding of BM cells to the antibodies in the microfluidic channels. Blocking reagent was washed three times by DPBS before being diluted 1:1 with 2% BSA-DPBS. Microfluidic processing steps remained the same with 1 µL/s as the sample introduction flow rate for cell capture, and 2 µL/s for the DPBS wash flow rate. For Fc blocking with adjusted flow rate, in addition to adding the Fc blocking agent, the sample introduction and DPBS wash flow rates were adjusted to 0.6 µL/s and 3 µL/s, respectively. The sample introduction flow rate was decreased to prolong the cell-antibody interaction time for higher capture. The DPBS wash flow rate was increased to enhance the removal of possible non-specifically captured cells. For each microfluidic device, 2 mL of diluted tumor-bearing BM was processed. For controls, MDA-MB-231-GFP cells were spiked into the naïve BM at a concentration of 1000 MDA-MB-231-GFP cells per five million naïve BM cells. These spiked-in samples were subjected to the same sample preparation and processing flow rates as their experimental counterparts and assessed for capture efficiency.

To determine the enrichment performance, the content captured in the devices was released using 0.25% trypsin with impulse. Released contents were subjected to flow cytometry analysis to detect live DTCs, defined as GFP^+^ events, and the results were used to determine the GFP^+^ frequency. The GFP^+^ frequency was derived from dividing the number of GFP^+^ events detected over the total number of cells detected in the BM sample, as given by the flow cytometry analysis. The fold enrichment was the factor by which the GEM had increased the GFP^+^ frequency in the BM sample.

### BM and blood samples collection

BM samples from healthy female donors were purchased from StemExpress (Folsom, CA, USA). Samples were certified to have $\geq 8\times 10^{7}$ total nucleated cells per 10-mL sample, with high cell viability. Blood samples from healthy donors were obtained from Innovative Research (Novi, MI, USA). Both healthy BM and blood samples were shipped overnight, received in EDTA-coated bottles, and processed on the day of receipt.

Clinical samples from patients were collected according to the protocol approved by the University of Florida Institutional Review Board (IRB) under IRB# 202000746. Written informed consent was obtained from each participant. The clinical samples used for this work cover from July 4, 2022 to January 25, 2023. One patient with TN breast cancer (GNV001-02) consented to the donation of BM aspirates and blood at two time points (pre-Cycle-1 as the baseline of the patient and post 4 cycles of treatment for comparison) to evaluate treatment effect. The treatment regimen for the patient consists of a 3-week cycle where 150 mg of sarilumab is administered subcutaneously (SQ) at the start of each cycle. Following a 3-day interval, the patient then begins a 14-day course of capecitabine, taken at a dose of 1000 mg/m^2^ daily. BM aspirates (10-20 mL) and blood samples (20-30 mL) were stored in BD Vacutainer EDTA tubes at ambient temperature, de-identified of patient information, and then transported to the research laboratory within one hour of collection.

### Processing of clinical samples

BM aspirates and whole blood samples were subjected to microfluidic processing on the day of sample collection. The samples were mixed with 2% BSA-DPBS at a 1:1 ratio to reduce the effects of blood viscosity variation among patients as previously described [23]. Four mL of the mixture (i.e., 2 mL of BM or whole blood mixed with 2 mL of 2% BSA-DPBS) was introduced through a functionalized GEM device at 1 µL/s. For comparison, each sample was processed by two GEM devices, one functionalized with anti-EpCAM antibodies, and the other functionalized with anti-EGFR antibodies. After sample introduction, nonspecifically adhered cells were washed out with DPBS (2 µL/s, 900 µL). Then, cells captured inside the device were immediately fixed with 4% paraformaldehyde (1 µL/s, 150 µL) for 10 min, followed by a DPBS wash (2 µL/s, 300 µL). Devices were placed in a humidified petri dish wrapped in aluminum foil and stored at 4 °C until immunocytochemistry staining.

To start the immunocytochemistry staining process, cell permeabilization were performed by introducing 0.2% Triton X-100 (1 µL/s, 150 µL) into the device, incubating for 10 min, and washing with DPBS (2 µL/s, 900 µL). Next, 2% BSA-DPBS (1 µL/s, 150 µL), used as a blocking buffer, was incubated in the device for 30 min. To detect the captured tumor cells in the device, a 100-µL antibody cocktail consisted of 2% BSA-DPBS, CD45-PE (20 µL, volume per test recommended by the manufacturer), and panCK-AF (final concentration 2 µg/mL) or CK-FITC (final concentration 2.5 µg/mL) was introduced into the device. The cocktail was incubated for 1 h at room temperature, followed by a DPBS wash (2 µL/s, 900 µL). Finally, the cells were incubated in 300 µM DAPI for 10 min and washed with DPBS (2 µL/s, 900 µL). Devices were stored in foil-wrapped humidified petri dishes at 4 °C until ready for fluorescence imaging (typically within two days).

The imaging of cells captured in microfluidic devices and cells in live cultures were conducted on the automated imaging system, BioTek Lionheart (Agilent, Santa Clara, CA, USA). The Lionheart setup used in this work featured three sets of LED cubes (365 nm, 465 nm, and 523 nm) and three filter cubes for multi-channel fluorescence image capture. The three filter cubes were DAPI (EX 377/50, EM 447/60), GFP (EX 469/35, EM 525/39), and red fluorescent protein (RFP, EX 531/40, EM 593/40). Images were processed and analyzed using the accompanying Gen5 Reader and Imager software. To determine the cell capture and release efficiencies, microfluidic devices were imaged on the BioTek and the number of cells immobilized in the device was enumerated using the Gen5 software. Similarly, for cell morphology and growth analysis, live MDA-MB-231-GFP cells in culture were imaged and then analyzed using the Gen5 software for parameters such as cell count and cell circularity. For the counting of CTCs and DTCs in clinical samples, devices were manually inspected using multi-channel fluorescence imaging at 20X. For this project, CTCs and DTCs were defined as having the phenotype of DAPI^+^panCK^+^CD45^-^ (**S5 Fig**).

### Statistical analyses

Statistical analysis was performed using GraphPad Prism 10 software. All Student’s t-tests were two-sided, and P values ≤0.05 (with 95% confidence intervals) were considered statistically significant for each specific statistical comparison (*P < 0.05. **P < 0.01, ***P < 0.001). In cases of multiple comparisons, adjustments were made using one-way ANOVA. Data are presented as mean ± SD (standard deviation) in the text and mean ± SEM (standard error of the mean) in figures. All experiments were repeated at least 3 times.

## Results

### DTC marker selection

Microfluidic enrichment efficiency is directly influenced by capture efficiency, which is dependent on two factors: (1) cell-capture agent affinity, and (2) cell-capture agent interaction frequency [24]. Previously, the GEM has been optimized to allow frequent interactions between the target tumor cells and the capture agents immobilized on the surface of the microchannels [15]. Now, we optimized cell-capture agent affinity by identifying target genes for the immunocapture of DTCs. First, we screened a panel of breast cancer drivers and markers gene that have been used for breast cancer detection (EGFR, EpCAM, KRT19, MUC1, IL6R, GLI3) [25] using a nested qPCR protocol against BM cells from healthy donors that had been spiked with the TN breast cancer cell line MDA-MB-231 or the ER^+^PR^+^ line MCF-7 at 1, 5, 50 spiked breast cancer cells in 1 million nucleated BM cells.

**Fig 1:**
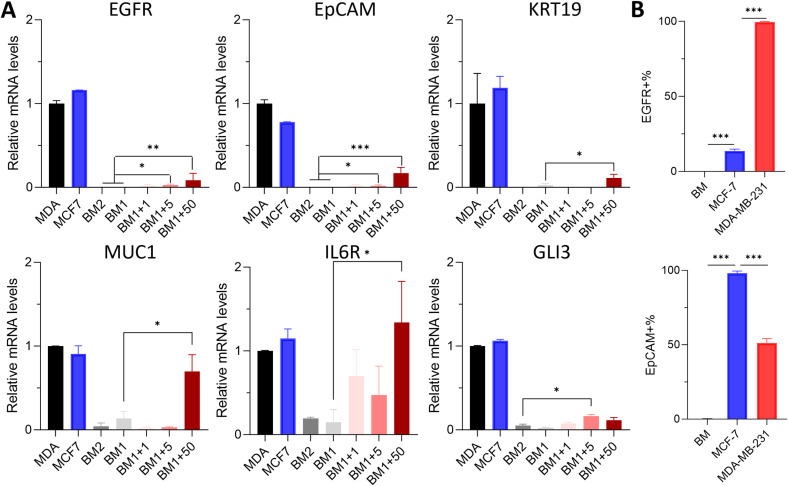
The mRNA levels of a gene panel were evaluated using nested qPCR and the affinity of MCF-7 and MDA-MB-231 cells to biotinylated anti-EpCAM and anti-EGFR antibodies as determined by flow cytometry. (A) Genes were all highly expressed in two cancer cell lines and barely expressed in BM samples. Specifically, there were no detectable levels of EGFR and EpCAM in both BM samples. MDA: MDA-MB-231; MCF7: MCF-7; BM1, BM2: bone marrow cells from healthy donors #1 and #2; BM1+1, +5, +50: 1, 5, 50 MDA-MB-231 cells were spiked into 1 million BM cells from healthy donor #1. (B) EGFR was highly expressed in MDA-MB-231 cells, an observation that aligned with the literature. Nucleated human BM cells were used as control. Streptavidin-PE was used to label biotinylated antibodies.

Our results showed that signals were successfully detected even at the lowest concentration of 1 MDA-MB-231 cell per 1 million BM cells, with signal intensity increasing proportionally to the number of spiked cells (**Fig 1A**). Importantly, EGFR and EpCAM exhibited no detectable signal in BM samples alone, highlighting their high specificity for cancer cells and demonstrating the sensitivity of our nested qPCR approach, particularly when 5 or more tumor cells were spiked into the sample. While other genes in the panel displayed lower specificity, with some mild expression in healthy donor BM samples, their differential expression was still detectable when the number of spiked cancer cells reached 50 per million BM cells.

In summary, this gene panel offers a reliable method for estimating the number of breast cancer cells mixed with bone marrow cells, with EGFR and EpCAM standing out as the most promising candidates due to their superior specificity and expression levels (**Fig 1A**). These findings align with previous research [25], though achieved here at significantly lower cell frequencies.

To assess the suitability of EGFR and EpCAM as cell surface targets for immunocapture, we evaluated their surface expression in MCF**-7** and MDA-MB-231 cells using flow cytometry (**Fig 1B**, **S6 Fig**). The expression of EGFR was almost universal in MDA-MB-231 cells (99.6 ± 0.5%), aligning with reported findings [26,27]. In contrast, MCF-7 cells exhibited much less EGFR expression, with only 13.6 ± 2.1% positivity [28]. EpCAM was highly expressed in MCF-7 cells (98.0 ± 2.7%), whereas MDA-MB-231 cells showed moderate surface expression, with a 51.2 ± 5.0% positive rate, corroborating prior reports [29].

Additionally, nucleated BM cells were analyzed and showed minimal expression of both EGFR and EpCAM, which is in line with the qPCR results presented in **Fig 1A**. Based on their expression profiles, both markers are suitable targets for capturing breast cancer cells in BM samples. MDA-MB-231 cells were selected for proof-of-concept experiments in the remainder of the study, given their suitability as a model for metastatic disease in mice.

### Microfluidic immunocapture of breast tumor cells

**Fig 2:**
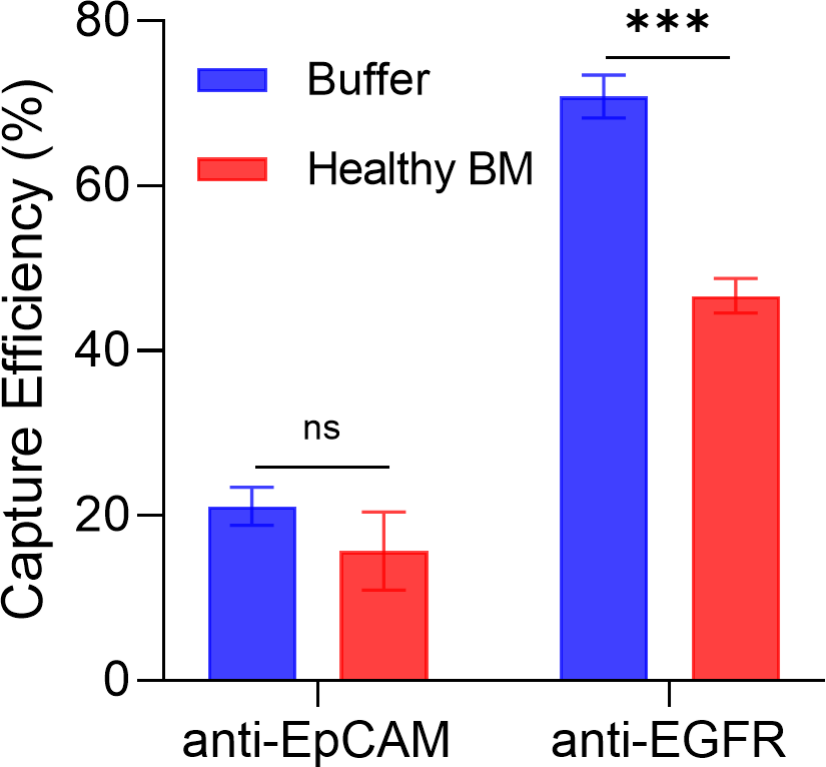
The cell capture efficiency of the GEM devices functionalized with either anti-EpCAM or anti-EGFR antibodies. Stained MDA-MB-231 cells were spiked in either 2% BSA-DPBS buffer or healthy BM aspirates.

To confirm the affinity between breast cancer cells and the capture agents (i.e., antibodies) in the microfluidic environment, we assessed the capture efficiencies of MDA-MB-231 cells spiked in 2% BSA-DPBS buffer and healthy BM. For buffer, the GEM delivered capture efficiencies of (21.1 ± 4.6)% using EpCAM antibodies and (70.8 ± 7.8)% using anti-EGFR (**Fig 2**). The inferior ability of anti-EpCAM to capture MDA-MB-231 cells fits along with the flow cytometry results (**Fig 1B**). These results highlight the necessity to carefully select the capture antibodies for affinity-based microfluidic platforms, and once again, confirm the important of choosing a suitable cell line for testing. For healthy BM, a similar difference in performance of the two capture antibodies was observed (**Fig 2**). However, compared to the buffer condition, the average capture efficiencies in the healthy BM condition decreased by ~5% for anti-EpCAM and ~24% for anti-EGFR. The decreased performance can be explained by the competition from non-target BM cells impeding the cell-capture agent interactions, the relatively higher viscosity of the BM, and the presence of cell clumps observed in the BM.

Overall, we chose anti-EGFR as the capture agent for the subsequent characterization of cell release methods and the enrichment of DTCs in BM (i.e., xenografted MDA-MB-231 cells). However, for the processing of clinical samples, GEM devices functionalized with either anti-EpCAM or anti-EGFR were used to ensure the capture of DTCs of varying surface markers since we do not know the true identity of tumor cells in clinical samples.

### Release of captured tumor cells from microfluidic device

**Fig 3:**
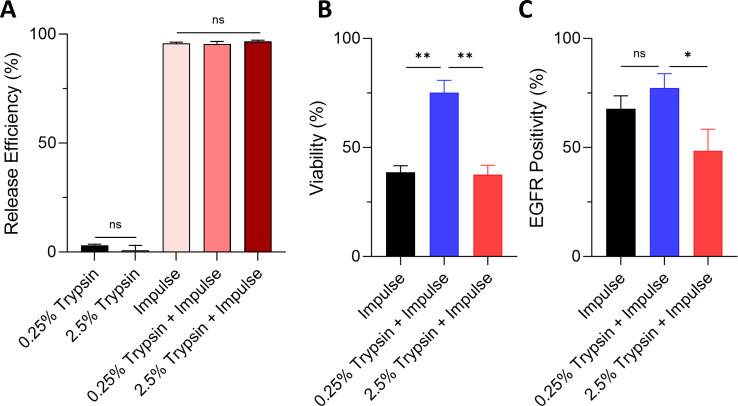
Release efficiency, viability and surface marker expression assessment of cells released by the three release methods: impulse, 0.25% trypsin with impulse, and 2.5% trypsin with impulse, in GEM devices coated with 10 ug/mL anti-EGFR antibodies. (A) Two chemical methods (e.g., 0.25% trypsin and 2.5% trypsin), one physical method (e.g., impulse), and two combinations were tested. The cell-dissociation agent trypsin only offered release efficiencies of less than 5%. However, the three methods utilizing mechanical means (impulse, 0.25% trypsin with impulse, and 2.5% trypsin with impulse) resulted in average release efficiencies of above 95%. (B) Viability of released cells divided by the viability of control cells. (C) EGFR positivity (i.e., percentage of cells showing positive EGFR expression by flow cytometry) of released cells divided by the EGFR positivity of control cells.

The contents captured inside the microfluidic device after processing must be efficiently retrieved to allow for robust downstream analysis (e.g., proteomic profiling, single-cell RNA sequencing, whole exome or genome sequencing) on the tumor cells of interest. To release cells captured in the device, we utilized chemical (i.e., trypsin) and mechanical (i.e., impulses) means. The use of either concentration of trypsin alone (0.25% trypsin or 2.5% trypsin) offered minimal release efficiency (**Fig 3A**). Remarkably, the incorporation of impulses for cell release (impulse, 0.25% trypsin with impulse, and 2.5% trypsin with impulse) delivered release efficiencies of above 95% (**Fig 3A**).

**Fig 4:**
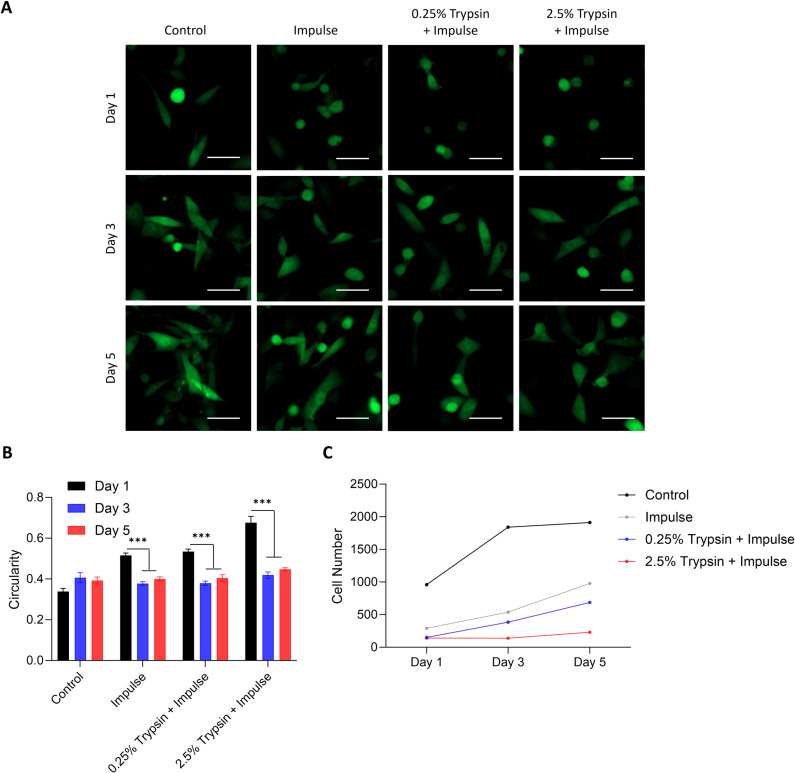
Morphologic assessment of MDA-MB-231-GFP cells released from EGFR-coated GEM microfluidic devices using release methods: (1) impulse, (2) 0.25% trypsin with impulse, and (3) 2.5% trypsin with impulse. (A) Fluorescence images were captured on day 1, day 3, and day 5. Scale bar is 50 µm. (B) Circularity analysis of cells at various locations of each culture well at the three timepoints. (C) Cell growth of the released cells analyzed at the same culture locations at the three timepoints.

In addition to the release efficiency, it is recommended that other quality metrics such as released cell viability, morphology, and functionality, be assessed to ensure the success of downstream analysis [30]. Thus, we then assessed the viability, surface marker expression, and morphology of the MDA-MB-231 cells released from the GEM devices using impulse, 0.25% trypsin with impulse, and 2.5% trypsin with impulse. We observed the highest cell viability for 0.25% trypsin with impulse at 75.23 ± 9.62% (**Fig 3B**). Cell viabilities for impulse and 2.5% trypsin with impulse were at 38.62 ± 5.17% and 37.52 ± 7.50%, respectively. Regarding EGFR expression, 67.67 ± 10.48% and 77.29 ± 11.45% of the viable cells released by impulse and 0.25% trypsin with impulse, respectively, maintained their EGFR expression. However, only 48.57 ± 17.05% of the viable cells released by 2.5% trypsin with impulse maintained theirs (**Fig 3C**), possibly due to excessive degradation of surface proteins caused by the high concentration of trypsin. As for morphology, the released cells of all three release conditions still retained their round shape typical of cells in suspension 1 day after cell release (**Fig 4A**). However, they adapted to their culture environment and regained the distinct spindle-shaped morphology observed in the control cells by day 3. We confirmed this observation via quantitative circularity analysis of fluorescence images. On day 1, the released cells possessed circularity values ranging from 0.50 to 0.74 (**Fig 4B**). By day 3, their circularity range had dropped to a range from 0.36 to 0.45, similar to that of the control cells. All three groups of released cells displayed a delayed growth rate in culture (**Fig 4C**), with 2.5% trypsin with impulse showing the lowest growth rate.

Collectively, the post-release cellular analysis results supported 0.25% trypsin with impulse as the optimal cell release method.

### Enrichment of DTCs in mouse BM

After the cell capture and release methods were established, we used them to study DTCs in mice as a proof-of-concept. We hypothesized that the microfluidic enrichment efficiency could be enhanced by optimizing the device operating conditions (**S4****A Fig**). For the standard normal flow rate condition, we observed a capture efficiency of 75.57 ± 7.12% (**S4****B Fig**), which was comparable to the efficiency obtained for capturing cells-in-buffer using the same operating conditions (**Fig 2**). Adding an Fc blocking agent to the BM sample to block the Fc receptors and discourage non-specific binding, and combining Fc blocking with flow rate adjustments, increased the efficiency to 85.75 ± 19.10% and 99.98 ± 1.54%, respectively (**S4****B Fig**). Thus, optimizing operating conditions led to a notable improvement in the average capture efficiency for tumor cells in naïve BM.

**Fig 5:**
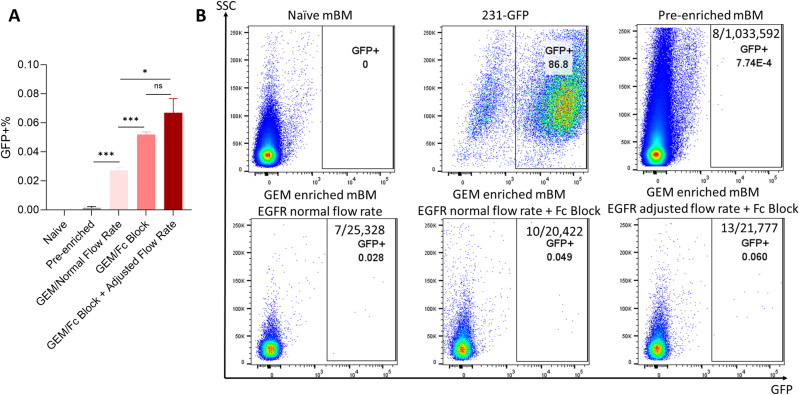
Naïve, metastatic BM and GEM processed cells were analyzed using flow cytometry. (A) Pre-enriched BM carried 0.0014 ± 0.0014% GFP+ cells. GEM with normal flow rate enriched the GFP^+^ cells to 0.0273 ± 0.0030% while GEM with Fc Block enriched to 0.0520 ± 0.0030%. The combination of gave the best enrichment to 0.067 ± 0.017%. (B) Representative images of flow cytometry. Naïve BM served as the negative control to set the gating threshold for GFP^+^ signal. The number of GFP^+^ cells detected over the total number of cells detected was expressed as the GFP^+^ frequency (%). The MDA-MB-231-GFP cells were used as the positive control.

We observed a similar trend in the enrichment efficiency for DTCs in mouse breast cancer model. Under normal flow rate conditions, the microfluidic device enriched DTC counts from a GFP^+^ frequency of (0.0014 ± 0.0014)% to (0.0273 ± 0.0030)%, representing approximately a 19.5-fold increase. Introducing Fc blocking during sample preparation drastically increased the enrichment to the range of (0.0520 ± 0.0030)%. With further adjustments to the sample introduction and wash flow rates on top of Fc blocking, we achieved the optimal performance at a GFP^+^ frequency of (0.067 ± 0.017)%, a 47.9-fold increase (**Fig 5A**).

In summary, our enrichment results pointed to the pivotal role of the Fc blocking agent in preventing the non-specific binding of BM cells to the capture antibodies in the microfluidic device, thereby enhancing enrichment efficiency [31]. Additionally, adjusting operating flow rates, alongside Fc blocking, further improved the capture efficiency (**Fig 5B**). The reduction in sample introduction flow rate likely extended the interaction time between target cells and antibodies, facilitating capture. However, this improvement was modest, as the reduced flow rate also increased the potential for non-target cells to adhere to microchannel walls, a phenomenon partially mitigated by an increased wash flow rate. Despite these challenges, the adjustments to sample preparation and microfluidic parameters demonstrated promise in achieving high enrichment efficiency.

### Detection of CTCs and DTCs in clinical samples

**Fig 6:**
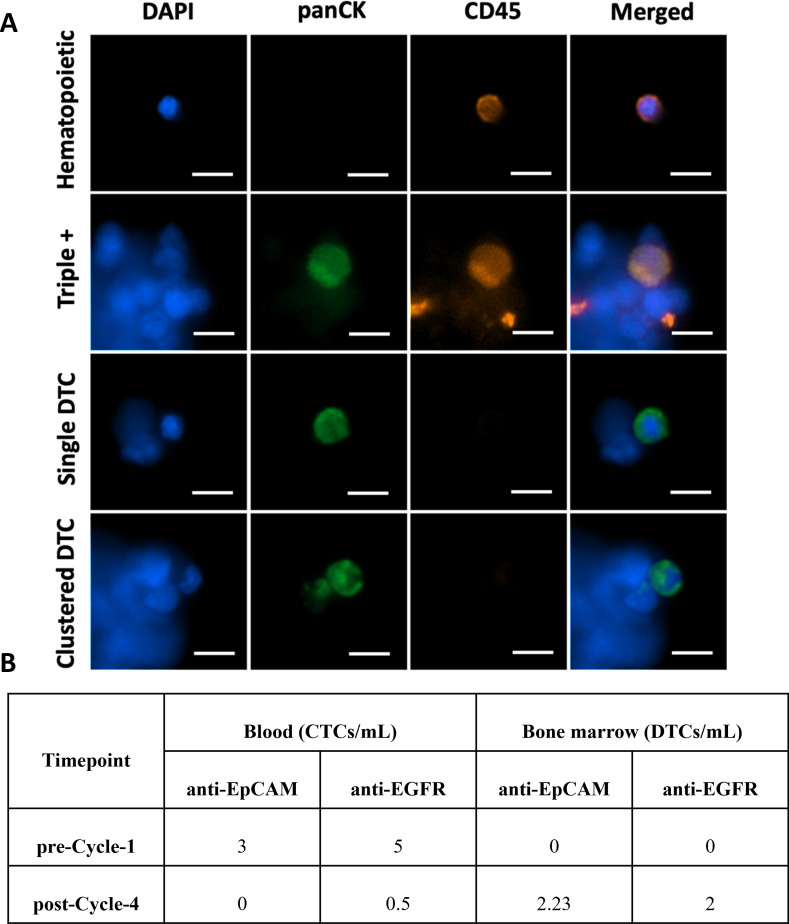
Nucleated cells found in a clinical BM sample. (A) Hematopoietic cells are defined as those with phenotype DAPI^+^panCK^-^CD45^+^. DTCs are defined by the phenotype DAPI^+^panCK^+^CD45^-^. Single DTC shows one nucleus (DAPI) housed within an intact cytoplasm (panCK). Clustered DTC shows two separate nuclei housed within two cytoplasmic panCK signals, one fragmented and one intact. Triple-positive cells (“Triple +”), defined as DAPI^+^panCK^+^CD45^+^, were also observed. Scale bars are 10 µm. (B) CTC and DTC numbers in samples obtained at two timepoints from the same patient. For each sample of whole blood or BM aspirates, 2 mL were processed. CTC and DTC enumeration were reported as CTC per mL and DTC per mL of sample processed, respectively, to facilitate comparisons between studies. Hence, to obtain the total number of CTC or DTC found in each sample, multiply each number seen in the table by two.

Lastly, we demonstrated the efficacy of the microfluidic enrichment method in human clinical BM samples. A BM aspirate and a blood sample were collected from patient GNV001-02 at timepoints pre-Cycle-1 and post-Cycle-4, and immediately subjected to microfluidic processing for CTC and DTC enumeration (**Fig 6**, **S7 Fig**). Note that we reported the tumor cell number per mL while many in the literature reported the number in 7.5 mL of blood (the volume used in CellSearch [32]). The baseline CTC (**S1** Table) and DTC (**S2** Table) counts were determined from processing healthy controls. Notably, we detected DAPI^+^panCK^+^CD45^+^ triple-positive cells in the clinical BM sample (**Fig 6A**). Such cells have been reported previously in CTCs and potentially linked to poor prognosis [33]. “Whether this holds true in DTCs” and “what the difference between triple positive DTCs and classical DAPI^+^panCK^+^CD45^-^ DTCs is” need to be further determined. Although the majority of detected CTCs/DTCs were singles, we also observed some clusters of two CTCs/DTCs (**S7** Fig and **Fig 6A**). Previously, others have reported the detection of CTCs as both single cells and multicellular clusters consisting between 2-50 CTCs [34–38]. Additionally, we observed an interesting phenomenon where CTC and DTC enumerations exhibited contradicting trends (**Fig 6B**). At pre-Cycle-1 timepoint, no DTCs were detected in either GEM device, while CTCs were detected at a low count. However, at post-Cycle-4, the DTC numbers had increased, while the CTC numbers had almost vanished. CTCs are well-recognized as prognostic indicators in early-stage breast cancer [39] and are often used to assess treatment responsiveness in advanced stages [40]. However, in this patient, the treatment response was paradoxical: although CTC counts decreased, the disease continued to progress. This suggests that while circulating tumor cells in the bloodstream were impacted, the increased DTC counts may indicate that tumor cells were able to survive and possibly thrive within the bone marrow or other distant sites. This adaptation could reflect a mechanism of resistance or evasion, allowing the tumor cells to bypass the effects of systemic therapy. Although the predictive value of DTCs is still a topic of debate [40,41], this case highlights the importance of further research to understand whether an increase in DTC numbers reflect individualized resistance patterns or represent a broader strategy for therapeutic escape.

## Discussions

The presence of DTCs in the BM and CTCs in the bloodstream has been frequently associated with late recurrences of breast cancer, yet few therapies have been developed to target these cells [4,42,43]. This underscores the significant unmet need to better understand DTCs in unraveling the complexities of metastasis and recurrence. Current research on DTCs often involves PCR amplification, which suggests these cells undergo clonal expansions and adapt to the BM environment [44]. Yet, their rarity poses significant challenges for capturing for further molecular characterization [2].

Microfluidic technologies offer potential solutions for efficiently enriching rare cells, with their high surface-area-to-volume ratio and precise control at micro- and nanoscale levels [30,45,46]. Effective cell capture and subsequent release without compromising cell integrity are crucial [30]. Recent advancements include surface modification strategies [47,48] and magnetically controllable interfaces for cell release [49]. Compared to these recent works, the release efficiency of 0.25% trypsin with impulse in our study is up to par, with a simpler and more economic strategy but lower cell viability.

It should be noted that there are a large body of literature on using microfluidics for CTC isolation as summarized in the reviews and books [50,51]. However, there is little work of using microfluidics on DTC analysis. The most of DTC research were performed using the CellSearch platform. Based on our literature search, there is no report yet on an immunoaffinity-based microfluidic platform optimized for isolating and releasing DTCs from BM.

Enzymatic digestion with trypsin is a common method for cell detachment in microfluidics [52–55]. In these work, trypsin-sensitivity of the cell binding mechanism or capture agent was credited for the high cell detachment [53–57]. In our work, the inability of both 0.25% trypsin and 2.5% trypsin to release captured cells may be explained by a trypsin-resistant transmembrane domain of EGFR that contained an EGF binding site [58]. The addition of impulses delivered higher release efficiencies. A possible explanation could be the desorption of avidin from the PDMS and glass substrates caused by the impulse-induced perturbations. In the meantime, we acknowledge that utilizing trypsin for cell detachment may not be ideal for certain downstream analyses, such as evaluating surface markers and conducting single-cell analyses. An alternative approach, such as mechanical picking of cells, might be more suitable in these cases.

The selection of EGFR and EpCAM as surface markers for isolating DTCs in our study represents a targeted approach, informed by their high prevalence in specific breast cancer subtypes. However, relying solely on these markers could limit the ability to capture a wider range of DTCs, particularly those that do not express EGFR or EpCAM at detectable levels. This limitation highlights a potential gap in our study, as DTCs expressing alternative or less common markers may be overlooked, resulting in an incomplete analysis in clinical applications. To address this, testing combinations of multiple surface markers could enhance capture efficiency and provide a more comprehensive view of DTC populations.

In addition to surface markers, incorporating other physical characteristics, such as cell size and deformability, could further refine our understanding of DTC heterogeneity. Exploring these factors may uncover new opportunities to optimize microfluidic isolation techniques and improve overall detection accuracy.

Another limitation of our study is the sample size of the clinical trials, which constrains the conclusiveness of our findings. Although bone marrow sampling to assess DTC status could be a valuable tool for monitoring disease progression and treatment response, it is often seen as too invasive, with many patients unwilling to undergo multiple bone marrow extractions. As a result, bone marrow sampling was not mandated for trial participation. To confirm the effectiveness and generalizability of our findings, a larger number of samples should be analyzed.

It should be note that there is no way to know the absolutely accurate number of CTCs or DTCs in our clinical samples, as in other clinical samples reported in the literature. However, this will not diminish the significance of the detected changes in the number of CTCs and DTCs over the cancer treatment. The CTC/DTC dynamics from cycle 1 (before treatment) to cycle 4 (after 8 weeks of chemotherapy) provides useful information associated with clinical intervention as other biomarker dynamics reported in the literature [59,60].

## Conclusions

The mechanisms underpinning breast cancer metastasis and the heterogeneity of therapeutic responses are intricately associated with the dynamics of DTCs. Despite DTCs being pivotal to understanding metastatic dissemination and recurrence in breast cancer, their scant prevalence coupled with the dense cellular milieu of the bone marrow poses significant challenges. Utilizing an advanced immunoaffinity-based microfluidic device, our investigation has successfully refined a methodology for the efficient capture, enrichment, and subsequent release of DTCs conducive to downstream molecular characterizations. By using EGFR and EpCAM as selective biomarkers, in conjunction with a hybrid approach of chemical and mechanical cell release methods, represents a significant advancement in DTC research. Our data from murine DTC models and human clinical samples corroborate the applicability and real-world translational potential of our approach. Interesting, our analysis of pre and post treatment blood and bone marrow samples reveals a potential dynamic interplay between the CTC and BM DTC populations that likely underlies the pathophysiology of minimal residual disease in breast cancer. As microfluidic technology continues to evolve, it promises to revolutionize the way we study, diagnose, and treat aggressive cancers like breast cancer.

## Supporting information

Fig S1 The geometrically enhanced mixing (GEM) microfluidic device. (PDF)

Fig S2 The cell release process. (PDF)

Fig S3 Enrichment of DTCs in mouse BM. (PDF)

Fig S4 Operating conditions for the microfluidic enrichment of DTCs in mouse BM using GEM devices. (PDF)

Fig S5 Representative images of cells captured from a healthy BM sample spiked with triple-negative breast cancer cells. (PDF)

Fig S6 Representative flow images related to Fig 1B. (PDF)

Fig S7 Nucleated cells found in a clinical blood sample. (PDF)

Table S1 Enumeration of DAPI+panCK+CD45- cells detected in healthy blood samples processed by GEM devices coated with anti-EpCAM or anti-EGFR antibodies. (PDF)

Table S2 Enumeration of DAPI+CK+CD45- or DAPI+panCK+CD45- cells detected in healthy bone marrow samples processed with GEM devices functionalized with either anti-EpCAM or anti-EGFR antibodies. (PDF)
