# Supplementary material for: Microfluidic isolation and release of live disseminated breast tumor cells in bone marrow
Source: PLoS One. 2025 Mar 12;20(3):e0319392. doi: 10.1371/journal.pone.0319392 (PMC11902295; doi:10.1371/journal.pone.0319392)
Supplement: Fig S1 — (PDF) [file pone.0319392.s001.pdf]

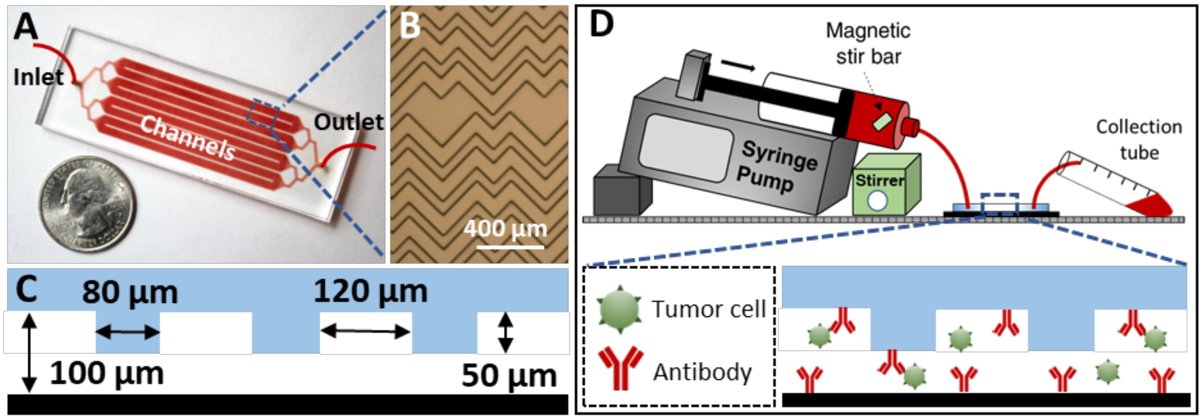

Figure S1. The geometrically enhanced mixing (GEM) microfluidic device. (A) The device, the size of a standard 3-inch by 1-inch glass slide, features one inlet, eight parallel microchannels, and one outlet. (B) Inside the microchannels are herringbone micromixers that induce chaotic mixing during laminar flow. (C) The dimensions of the micromixer groove features are 100 µm for channel depth, 50 µm for groove depth, and 120 µm for groove width. (D) The standard setup for passing a sample through a microfluidic device. In this setup, a syringe pump was used to deliver the sample at a specified flow rate, a magnetic stir bar placed inside the sample and a stir plate placed below the syringe were used to prevent cells from settling down, and a tube was attached to the outlet of the device to collect waste or other released contents. When the inside of the microchannels was functionalized with antibodies, target tumor cells were captured via immunoaffinity.
