# Supplementary material for: Microfluidic isolation and release of live disseminated breast tumor cells in bone marrow
Source: PLoS One. 2025 Mar 12;20(3):e0319392. doi: 10.1371/journal.pone.0319392 (PMC11902295; doi:10.1371/journal.pone.0319392)
Supplement: Fig S2 — (PDF) [file pone.0319392.s002.pdf]

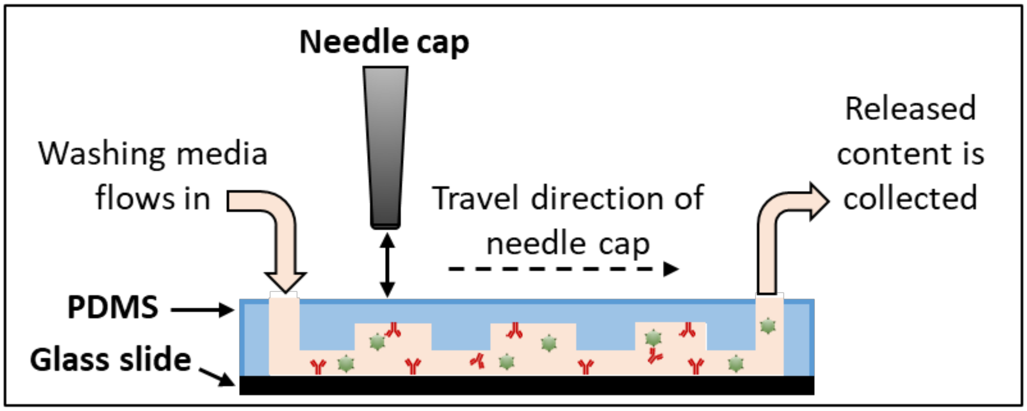


**Figure S2. The cell release process.** Cell dissociation chemical, trypsin, was incubated inside the microchannels, before fully supplemented cell culture media was pumped through the device at a high flow rate to wash out the detached cells. To enhance the release efficiency, a needle cap was used to provide additional impulse to the device during washing. Impulse was provided by repeatedly tapping the needle cap on the PDMS side of the device, directly on top and along the length of each microchannel. One round of tapping consisted of 12 taps, at a speed of 180 taps per minute, in the indicated travel direction. During the ~13 min of the wash step, each microchannel received two rounds of tapping (24 taps) every 2 min.
