# Supplementary material for: Microfluidic isolation and release of live disseminated breast tumor cells in bone marrow
Source: PLoS One. 2025 Mar 12;20(3):e0319392. doi: 10.1371/journal.pone.0319392 (PMC11902295; doi:10.1371/journal.pone.0319392)
Supplement: Fig S3 — (PDF) [file pone.0319392.s003.pdf]

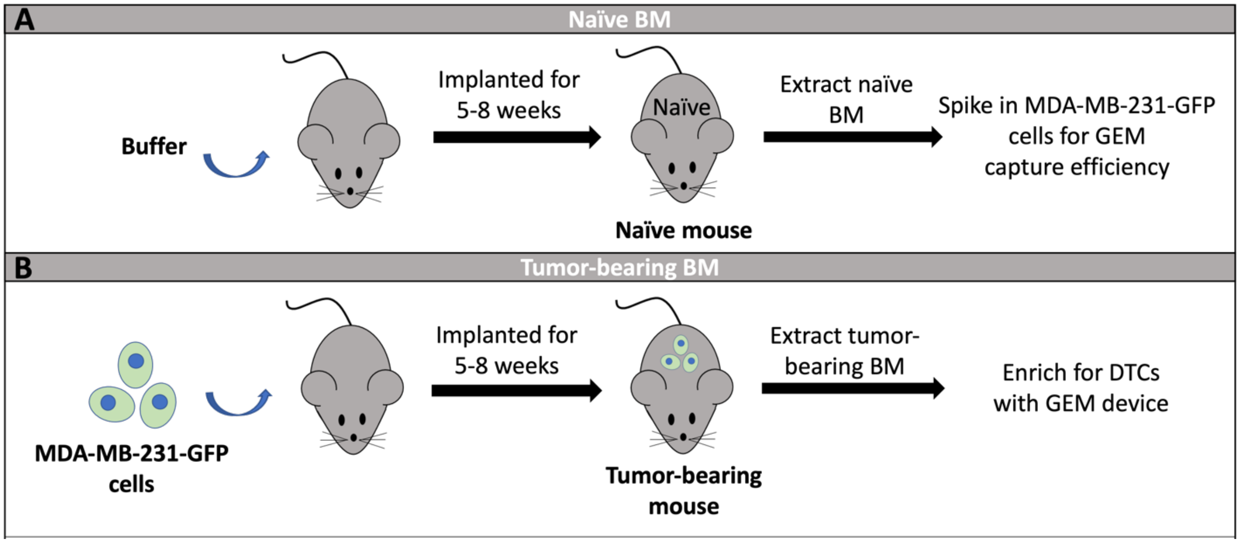

Figure S3. Enrichment of DTCs in mouse BM. (A) For control, a buffer was injected into the mammary fat pad of immunocompromised mice (n=5). After five to eight weeks, the mice were sacrificed, and the BM cells were harvested. To ensure acceptable capture efficiencies in the devices, MDA-MB-231-GFP cells were spiked into the naïve BM and processed with a microfluidic device. (B) MDA-MB-231-GFP cells were injected into immunocompromised mice (n=10). After five to eight weeks of implantation, tumor-bearing BM were collected for microfluidic processing. Cells captured inside the device were released by 0.25% trypsin with impulse.
