# Supplementary material for: Microfluidic isolation and release of live disseminated breast tumor cells in bone marrow
Source: PLoS One. 2025 Mar 12;20(3):e0319392. doi: 10.1371/journal.pone.0319392 (PMC11902295; doi:10.1371/journal.pone.0319392)
Supplement: Fig S4 — (PDF) [file pone.0319392.s004.pdf]

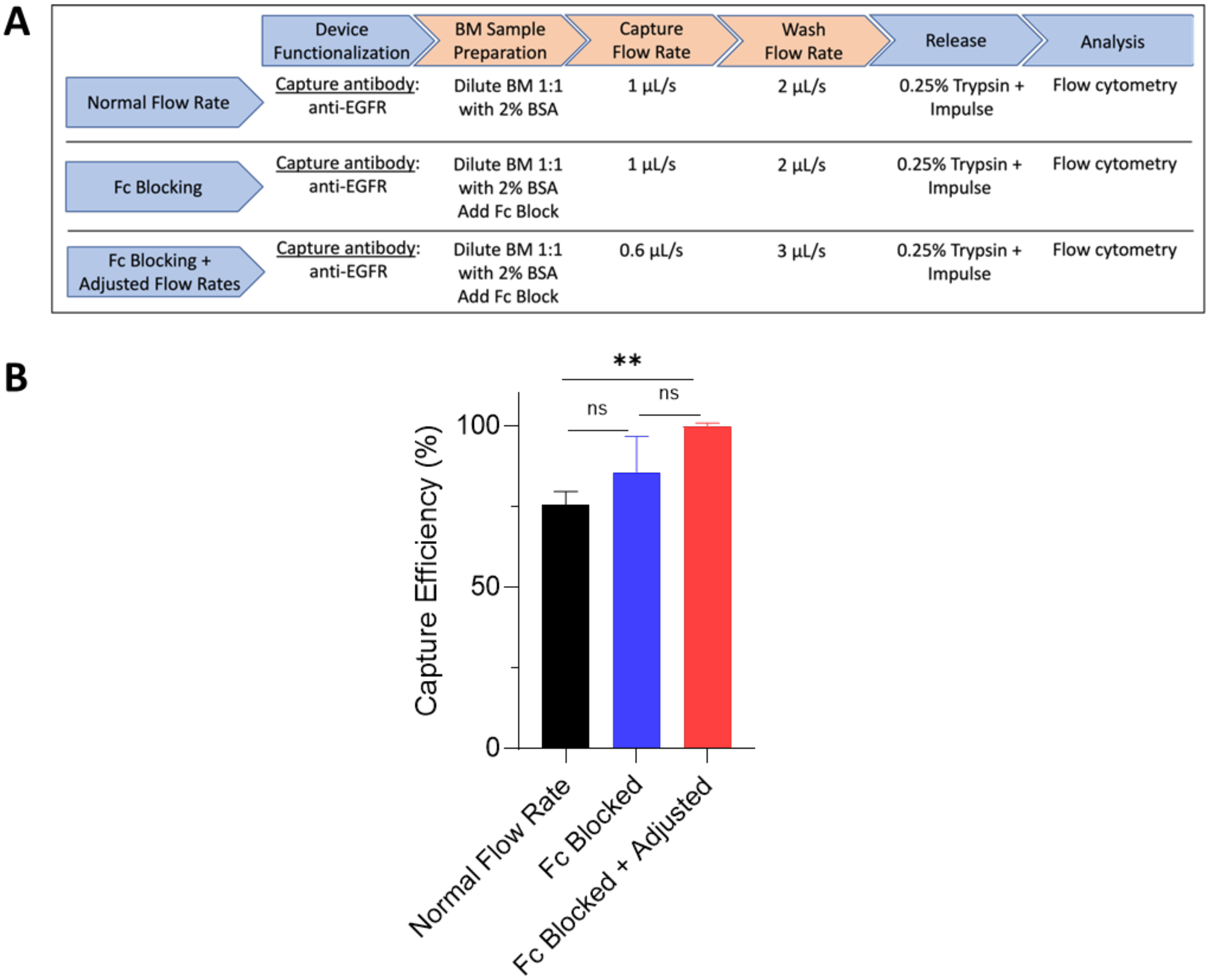

Figure S4. Operating conditions for the microfluidic enrichment of DTCs in mouse BM using GEM devices. (A) Three conditions with different sample preparation and processing flow rates were tested: (1) normal flow rate, (2) Fc blocking, and (3) Fc blocking with adjusted flow rates. (B) Capture efficiency of GEM devices (n = 3) functionalized with anti-EGFR antibodies for processing control mouse BM samples. MDA-MB-231-GFP cells were spiked into naïve BM for processing with microfluidic devices to ensure acceptable performance during the processing of BM samples.
