# Supplementary material for: Microfluidic isolation and release of live disseminated breast tumor cells in bone marrow
Source: PLoS One. 2025 Mar 12;20(3):e0319392. doi: 10.1371/journal.pone.0319392 (PMC11902295; doi:10.1371/journal.pone.0319392)
Supplement: Fig S5 — (PDF) [file pone.0319392.s005.pdf]

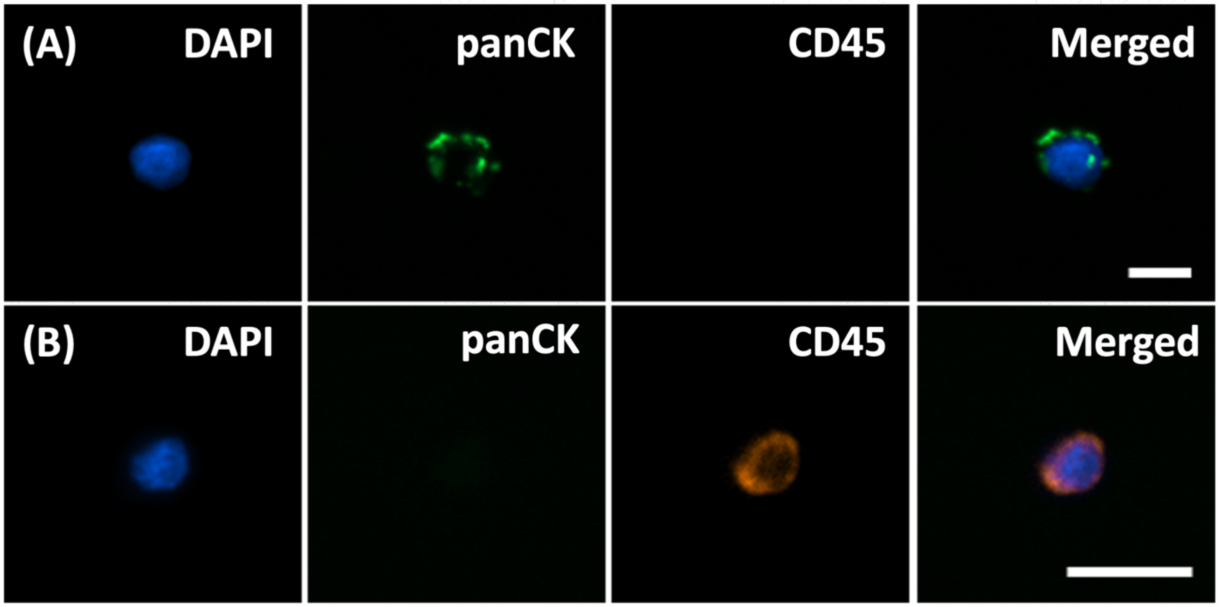

Figure S5. Representative images of cells captured from a healthy BM sample spiked with triple-negative breast cancer cells. (A) An MDA-MB-231 cell with $DAPI^+$, $panCK^+$, and $CD45^-$. (B) A white blood cell with $DAPI^+$, $panCK^-$, and $CD45^+$. Scale bars are 20 µm.
