# Supplementary material for: Microfluidic isolation and release of live disseminated breast tumor cells in bone marrow
Source: PLoS One. 2025 Mar 12;20(3):e0319392. doi: 10.1371/journal.pone.0319392 (PMC11902295; doi:10.1371/journal.pone.0319392)
Supplement: Fig S6 — (PDF) [file pone.0319392.s006.pdf]

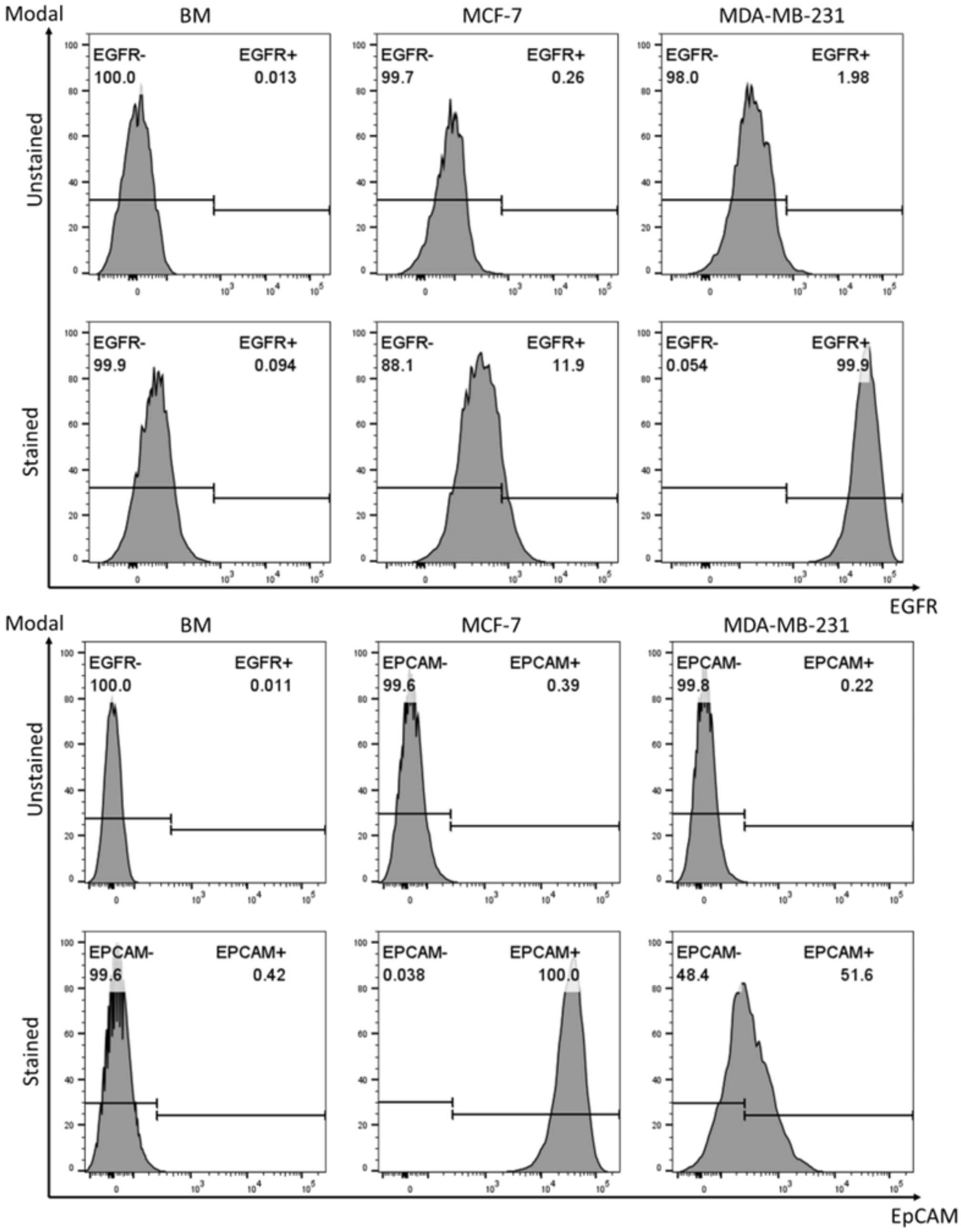

Figure S6. Representative flow images related to Figure 1B. The cutoff was based on the maximum expression of unstained control cells for each antibody. For EGFR, we utilized unstained MDA-MB-231 to set up the gate, and MCF-7 was used to set up the gate for EpCAM.
