# Supplementary material for: Microfluidic isolation and release of live disseminated breast tumor cells in bone marrow
Source: PLoS One. 2025 Mar 12;20(3):e0319392. doi: 10.1371/journal.pone.0319392 (PMC11902295; doi:10.1371/journal.pone.0319392)
Supplement: Fig S7 — (PDF) [file pone.0319392.s007.pdf]

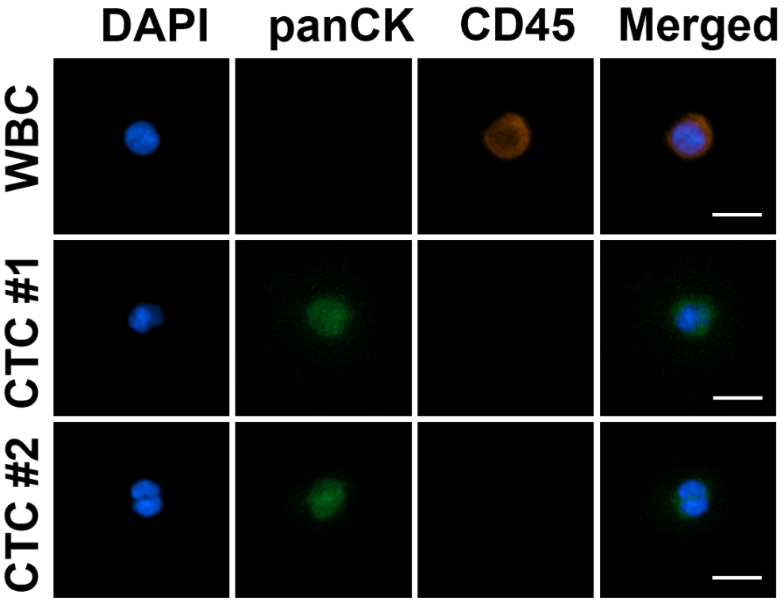

Figure S7. Nucleated cells found in a clinical blood sample. White blood cells (WBCs) were defined as those possessing phenotype $DAPI^{+}panCK^{-}CD45^{+}$. Circulating tumor cells (CTCs) are defined by the phenotype $DAPI^{+}panCK^{+}CD45$-. CTC #1 shows one nucleus (DAPI) housed within one intact cytoplasm (panCK). CTC #2 shows two separate nuclei, suggesting a cluster of CTCs. Scale bars are 10 μm.
