# Supplementary material for: Microfluidic isolation and release of live disseminated breast tumor cells in bone marrow
Source: PLoS One. 2025 Mar 12;20(3):e0319392. doi: 10.1371/journal.pone.0319392 (PMC11902295; doi:10.1371/journal.pone.0319392)
Supplement: Table S1 — (PDF) [file pone.0319392.s008.pdf]

Table S1. Enumeration of $DAPI^{+}panCK^{+}CD45^{-}$ cells detected in healthy blood samples processed by GEM devices coated with anti-EpCAM or anti-EGFR antibodies.

Capture Antibody	Healthy Donor Blood Experiment	$DAPI^{+}panCK^{+}CD45^{-}$ cells/mL
Anti-EpCAM	1	0
	2	0
	3	0
Anti-EGFR	1	0
	2	0
	3	0
