# Supplementary material for: Microfluidic isolation and release of live disseminated breast tumor cells in bone marrow
Source: PLoS One. 2025 Mar 12;20(3):e0319392. doi: 10.1371/journal.pone.0319392 (PMC11902295; doi:10.1371/journal.pone.0319392)
Supplement: Table S2 — (PDF) [file pone.0319392.s009.pdf]

Table S2. Enumeration of $DAPI^{+}CK^{+}CD45^{-}$ or $DAPI^{+}panCK^{+}CD45^{-}$ cells detected in healthy bone marrow samples processed with GEM devices functionalized with either anti-EpCAM or anti-EGFR antibodies. For each sample, 1 to 2 mL (depending on the volume available) were processed by the microfluidic device.

Capture Antibody	Healthy Donor BM Experiment	$DAPI^{+}CK^{+}CD45^{-}$ cells/mL
Anti-EpCAM	1	0.77‡
	2	0
Anti-EGFR	1	0
	2	0

‡ One $DAPI^{+}CK^{+}CD45^{-}$ cell was detected in this 1.3-mL sample, resulting in 0.77 cells/mL. The higher value of 0.77 cells/mL was taken as the baseline DTC/mL value for clinical BM samples processed by GEM devices with anti-EpCAM antibodies.
